# News media coverage of LGBT identities over 10 years in a 400-million-word corpus

**DOI:** 10.1371/journal.pone.0300385

**Published:** 2024-04-10

**Authors:** Reuben Ng, Ting Yu Joanne Chow, Wenshu Yang

**Affiliations:** 1 Lee Kuan Yew School of Public Policy, National University of Singapore, Singapore, Singapore; 2 Lloyd’s Register Institute for the Public Understanding of Risk, National University of Singapore, Singapore, Singapore; University of Perugia: Universita degli Studi di Perugia, ITALY

## Abstract

**Background:**

This study is the first to analyze LGBT portrayals in a news media dataset over a decade (2010–2020). We selected Singapore as a country of interest, emblematic of a nation grappling with state-encouraged heteronormativity and a remnant colonial law against homosexuality (377A), fraught with calls for its repeal that was only enacted in 2022. Our study is interested in this period bookended by challenge and change, particularly in newspaper portrayals of LGBT narratives. Newspapers are an important source of current information and have the power to shape societal perceptions. We lay the groundwork and provide a framework to analyze news media narratives of other Commonwealth nations with colonial pasts and inherited laws criminalizing LGBT communities.

**Objectives:**

This study analyzes LGBT portrayals in a 400-million-word news media dataset over a decade (2010–2020). First, we aimed to track the volume of LGBT media coverage over time and elucidate differences in coverage of different identity markers. Second, we aimed to track sentiments on LGBT portrayals. Third, we aimed to track salient narratives circulated about LGBT stories.

**Methods:**

The study leveraged a 400-million-word corpus from news media in Singapore, identifying the following target keywords: *LGBT*, *Lesbian*, *Gay*, *Bisexual*, *Transgender*, *Pink Dot* (a local Pride event), *377A*. First, coverage volume was tracked using annual changes in keyword mentions per million, elucidating differences in coverage of different sub-groups. Second, sentiment analysis on a valence scale was conducted on LGBT collocates. Third, we distilled salient narratives about LGBT identities using thematic labelling of top-frequency collocates.

**Results:**

First, overall coverage of LGBT steadily increased over the decade, though Gay identities evidenced asymmetrical coverage—outstripping ‘Bisexual’ keywords by seven times, ‘Lesbian’ by four, ‘Transgender’ by two. Second, sentiment scores for *Pink Dot (a local pride event)* were most positive; *Lesbian*, *Gay*, *LGBT*, *Transgender* were neutral; *Bisexual* and *377A* dipped slightly negative. Third, topics differed across the four identities: uniquely, ‘Lesbian’ collocates related to sensationalized cinema; ‘Gay’ about hate crimes; ‘Bisexual’ about population surveys; ‘Transgender’ about challenges (transitioning, alienation, suicide).

**Conclusions:**

Practically, we presented a decade-long barometer of LGBT sentiments and themes on a national level, providing a framework to analyze media for more effective communication strategies—applicable to Commonwealth countries with similar inherited colonial laws. Salient repetition through media association may unwittingly frame certain issues negatively; caution is prudent in representing each sub-group adequately, rather than portraying the LGBT identity as monolithic.

## Introduction

While there has been invigorated interest in LGBT-related research topics within academia [1], publications have mostly delved into focus-group data or the analysis of a specific issue within the demographic [2, 3]. Comparatively few studies have taken a big data approach to systematically analyze online mainstream news media for overall coverage of LGBT narratives, with research especially lacking in Asian nations [4], which may be useful for communication strategists or policymakers in evaluating the impact of media messaging on a population’s attitudes toward non-heterosexuality [5]. In this study, we leveraged a database of over 400 million words over the span of 11 years from 2010–2020 (henceforth referred to as the 2010s decade as shorthand), to identify trends in mainstream media coverage about the LGBT demographic.

Specifically, our study focused on news media coverage of this demographic over the decade. Within Singapore, this demographic occupies a curious societal grey-area, where the state encourages heteronormativity, but does not fully condemn homosexuality. While a law against homosexuality—Penal Code Section 377A (colloquially, 377A), prohibiting homosexual male partners from consensual sexual acts—has existed since the country’s independence in 1965 and only repealed in 2022, the law was tenuously-enforced, existing to placate conservative swathes of society [6, 7]. In 2007, the nation’s Prime Minister conceded that 377A was not to be proactively enforced, but would remain unrepealed given its status as societal lightning rod [8]. Despite this, LGBT groups continued to face polarized resistance [9, 10] amidst a struggle to repeal 377A [11] and against a backdrop of state-encouraged heteronormativity—in which only straight couples are allowed to marry, apply for public housing—mandating the careful negotiation LGBT individuals in a liminal societal space [12].

Within a culturally conservative Southeast Asia, political and social stressors affect LGBT individuals more than cisgender or heterosexual people: a systematic review of quantitative studies about LGBT Southeast Asians indicate a higher prevalence of mental health difficulties [13], and suicidal ideation in non-heterosexual Singaporean men [14]. Ethnographic interviews with gay Singaporean men revealing hesitance to ‘come out’ as gay due to fear of prosecution and discrimination [15]; and ambivalence at occupying societal marginality [16].

Such negativity is compounded by representations in media—especially in traditional offline media—that portray LGBT identities as one-dimensional, often ignoring many LGBTQ sub-groups in favor of other, more prominent ones (e.g., gay identities) [17]. These trends in media representations carry implications in socially constructing and shaping the way LGBT identities are perceived by the wider public [18], in many cases implicitly constructing them as ‘other’ [19]. Evidence suggests that mainstream media plays a mediating role in exposing LGBT stories to individuals unfamiliar with, or without LGBT acquaintances in their social circles, mainstreaming themes like tolerance and acceptability [20], making the mass-mediated medium important to study [21]. Media scholars also highlight the impact of news media on public perceptions, particularly as certain narratives are foregrounded via agenda setting [22], mainstreaming and magnifying certain broad narratives [23], thus shaping perceptions on social issues [24]. As such, news media must be interrogated for balanced and socially-responsible reporting on LGBT issues due to its mediated impact on community cohesion [25] and perceptual legitimacy [26].

The significance of such an undertaking is underscored by the higher susceptibility of LGBT populations to social inequality [27], discrimination [28], and hate speech [29]; impacting mental and physical health: increased risk factors for mental health issues [30] and healthcare hurdles [31]. Despite these risk factors, research into this demographic has only gained more traction in recent years [1]; such pertinent social issues present impetus for researchers to address, and for policymakers to pay crucial attention to.

### Research aims and objectives

With an established context highlighting existing issues of marginality of LGBT individuals in Singapore, the mediating role of news media in constructing LGBT identities, and the higher negative health outcomes associated with this marginal demographic, this study conducted an exploratory investigation into three metrics to understand mainstream news media narratives of LGBT in Singapore using big-data corpus analysis. First, we tracked the coverage size of LGBT topics over the decade to establish overall trends in mainstream media foci, and to elucidate if any volumetric differences existed in the coverage of different identity markers. Second, we tracked the average sentiment score of LGBT collocates. Third, we tracked the thematic content of top-frequency LGBT collocates, highlighting the salient narratives circulated about LGBT identities.

## Materials and methods

### Dataset pre-processing

All news articles from January 1, 2010, to December 31, 2020, were extracted from the News on the Web Corpus, culminating a dataset of 409,621,090 words. The corpus contains data from web-based newspapers and magazines from 2010 to the present time. Of this dataset, all news articles containing the following keywords were selected as part of the corpus, and split into the following categorial brackets: LGBT identities in general (*LGBT*, *LGBTQ*, *GLBT*, *Homosexual*, *Genderqueer*, *Pansexual*), Gay identities (*Gay(s)*, *including Gay man/men*), Lesbian identities (*Lesbian(s)*), Bisexual identities (*Bisexual(s)*, including *Bisexuality*), Transgender identities (*Transgender*, *Transsexual*, *Transvestite*—a term now considered derogatory in modern social contexts (barring reclamation by the community) but included in the study due to its common use in older news articles*—Trans Man*, *Trans Woman*), Pride Event Pink Dot (*Pink Dot*, *Pinkdot*), and Anti-homosexual law 377A (*377A*, including *Penal Code 377A*, *Section 377A*).

### Collocate selection, sentiment coding and thematic analysis

All articles in the selected corpus were subsequently pre-processed by tagging for their word, lemma, and part-of-speech; using these tags, the dataset was cleaned by filtering and removing redundant parts of speech, including—but not exhaustively—proper nouns; personal pronouns like *I*, *he*, *she*, *they*, *them*, *him*, *her*, *us*, *we*, *you*; subordinating conjunctions like *if*, *because*, *unless*, *so*, *for;* coordinating conjunctions like *and*, *or;* articles like *a*, *an*, *the;* forms of ‘be’ (finite/infinitive) like *is*, *are*, *was*, *were*, *be*, *being*, *been*, *am;* forms of ‘have’ like *have*, *had*, *having*, *has*. This filtering process ensured that content-heavy words (i.e., adjectives, nouns, verbs, adverbs) remained, ensuring that subsequent collocate analysis would yield meaningful results.

Our first investigative aim pertained to tracking the coverage size of LGBT topics in the decade. The volume of the following LGBT keyword categories was tracked in mentions per million, per annum from 2010 to 2020: *LGBT*, *Lesbian*, *Gay*, *Bisexual*, *Transgender*; to obtain a big-picture view of changes over time, and establish overall trends in mainstream media foci, and whether any coverage bias (i.e., differences in frequency of mentions indicating a skew in publication focus toward each sub-group) was present in representing various LGBT identity markers.

Our second and third investigative aim involved the analysis of LGBT collocates (i.e., words that co-occurred most frequently and in proximity to our target LGBT keywords). Specifically, our second aim focused on sentiment analysis of LGBT collocates, and the third aim on thematic analysis of LGBT collocates. To obtain this data from our corpus, we shortlisted all LGBT collocates based on the following criteria: First, collocates had to occur in a relevant context within a lexical proximity of ±6 words, coming either before or after the target LGBT keyword.

For instance, a sample of a news article from our selected corpus contained the following line:

*She cited the example of families bullying LGBTQ individuals with verbal abuse*, *harassment*, *and the performance of religious rituals and prayers over the individual to “correct” their sexuality*. [32] *(Published October 14*, *2019)*

Within this sample text, the data pre-processing stage removed personal pronoun *she*, determiner *the*, prepositions *of* and *with*, conjunction *and*; leaving meaningful collocates like *cited*, *example*, *families*, *bullying*, *individuals*, *verbal*, *abuse*, *harassment*, *performance*, *religious* within the lexical proximity (±6 words) of the “LGBT” target keyword.

Collocates also had to have a mutual information score of ≥ 1.5, indicating a stronger association with our target word relative to other words in the corpus, and was calculated using the following formulae: $\frac{\log\left[\frac{C*SizeCorpus}{A*B*Span}\right]}{\log\;2}$, where ‘A’ denoted the possibility of the target word A appearing, i.e. the frequency of our target LGBT keyword; ‘B’ denoted the possibility of the collocate B appearing, i.e. the frequency of the LGBT collocate; ‘C’ denoted the possibility of words ‘A’ and ‘B’ appearing together, i.e. the frequency of collocate ‘B’ appearing near the target word ‘A’; Size Corpus denoted the size of our corpus, i.e. the number of words; and Span had the value of 12; fulfilling the criteria of 6 words to the left and 6 words to the right of our target LGBT keyword. In short, the higher the MI value, the closer the relationship between our target LGBT keyword (A) and collocate (B); suggesting a higher chance of appearing together rather than separately, indicating semantic proximity. The formula is as follows:

$$\frac{C*SizeCorpus}{A*B}=\frac{C}{SizeCorpus}÷\frac{A*B}{SizeCorpus*2}=\frac{p(AB)}{p(A)*p(B)}$$

This process culminated in 8295 unique collocates that met the selection criteria.

Upon qualifying for our selection criteria, these collocates were rated on a valence scale of 1 (very negative) to 5 (very positive) by two independent raters; a validated methodology in tracking sentiment trends over time [33–38]. For instance, in the above example text, words like *bullying*, *abuse*, *harassment* were rated 1 (very negative). Other examples of words in the corpus that were rated 1 were: *shocked*, *disappointed*, *backlash*, *disparagingly*, *scorned*, *hate-motivated*, *degrading*, *cyber-bullying*. Words like *co-parenting*, *reassignment*, *masculine*, *autobiographical*, *societal*, *oft-discussed*, *duality*, *transitioning* were rated 3 (neutral). Words like *affirming*, *assured*, *empowered*, *commendation*, *praiseworthy*, *inclusivity*, *encouragingly*, *upliftment* were rated 5 (very positive). These collocate sentiment values were then used to produce an overall sentiment score—which were averaged and normalized by its frequency of appearance in the corpus.

The results from this process were used to fulfil our second investigative aim of tracking the sentiment score of LGBT collocates. Specifically, each category of keywords *LGBT*, *Lesbian*, *Gay*, *Bisexual*, *Transgender*, *Pink Dot* (the local Pride event), *377A* contained a sub-set of associated collocates that were rated on this valence scale.

Our third investigative aim of tracking the thematic content associated with LGBT identities was achieved by obtaining a sample of 100 top-frequency collocates from each keyword category: *LGBT*, *Lesbian*, *Gay*, *Bisexual*, *Transgender*, with each identity marker generating a different set of highest-frequency collocates unique to the category. This list was thematically labelled by two independent researchers to track salient narratives encoded in mainstream media. All data pre-processing, text analytics and statistical analyses were conducted in Python 3.7 and Origin Pro 2019b.

## Results

### Coverage size of LGBT categories over a decade

**Fig 1:**
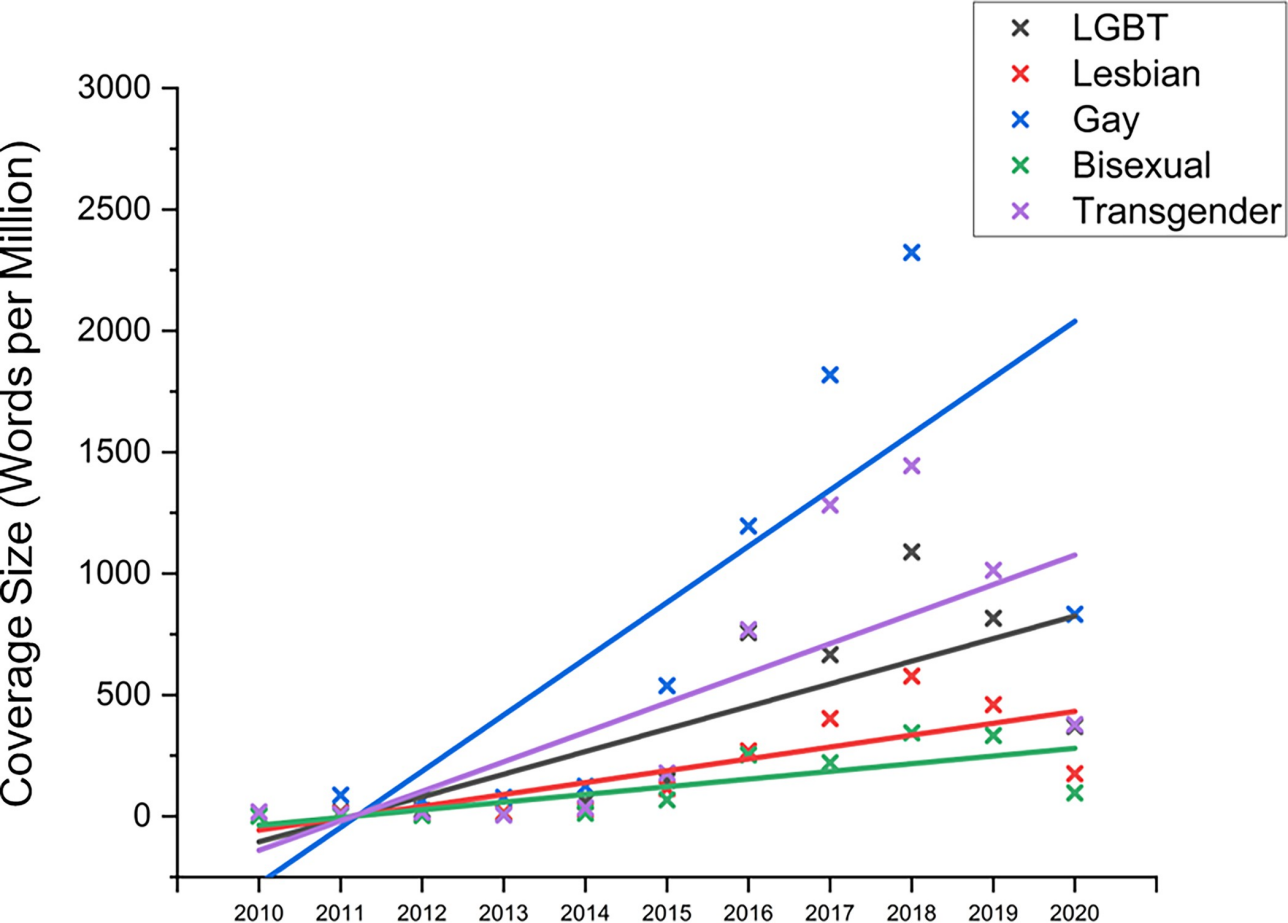
Coverage size over time of LGBT keywords in Singapore news media over a decade (2010–2020). Overall increase in mainstream media mentions of LGBT keywords over the decade from 2010 to 2020. Coverage was skewed in favor of keywords related to gay identity markers, followed by transgender identity markers, and with lesbian and bisexual identity markers lagging in comparison.

**Fig 2:**
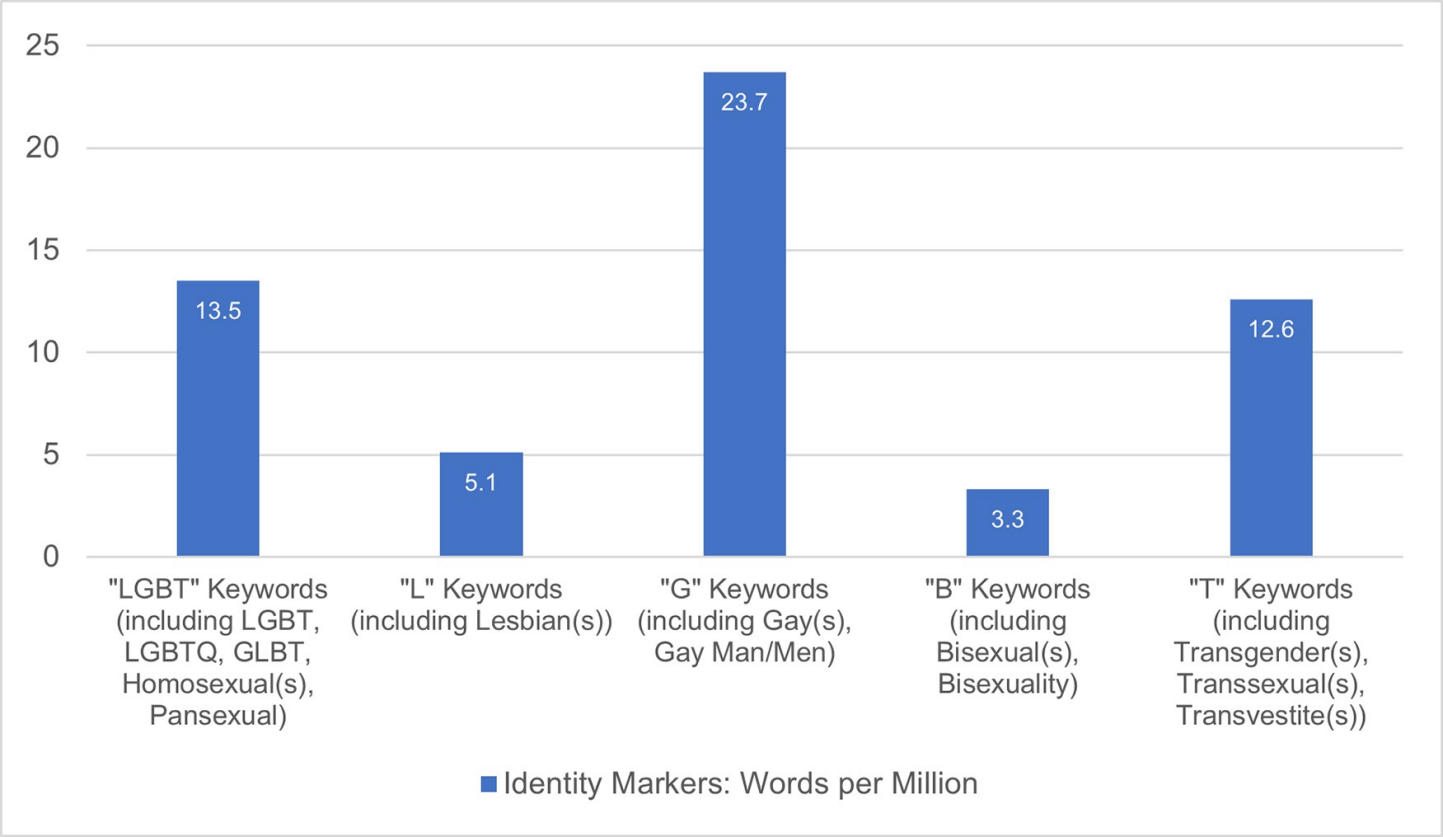
Comparative disparity in overall coverage size of LGBT keywords in news media over a decade from 2010 to 2020. Over the span of the decade (2010–2020), comparative disparities in coverage volume were evident. On average, Gay identity markers (keywords including *‘gay(s)’*, *‘gay man/men’*) were mentioned at a higher frequency than other identity markers; with coverage of this group outstripping Bisexual keywords (including ‘*bisexual(s)’*, *‘bisexuality’*) by seven times, Lesbian (keywords including *‘lesbian(s)*’) by four, Transgender (keywords including *‘transgender(s)’*, *‘transsexual(s)’*, *‘transvestite(s)’*) by two, even outnumbering general keywords denoting LGBT identities as a collective whole (including keywords *‘LGBT(Q)’*, *‘GLBT’*, *‘homosexual(s)’*, *‘pansexual(s)’*).

Over the 2010–2020-decade, coverage (average keyword mentions per million) about the LGBT demographic increased steadily over time in the 400-million-word news media dataset (Fig 1). All the respective trends were statistically significant at *p* < .01 (taking into account multiple comparisons, using the Bonferroni correction). Slope coefficients were above zero, indicating that the mean increase in coverage of respective keywords were positive, and that this gradual year-on-year slope of increase was significant. It was notable, however, that ‘Gay’ keywords evidenced the highest coverage over the decade, compared to other LGBT keywords (Fig 2).

### Description of sentiment scores of LGBT categories

**Fig 3:**
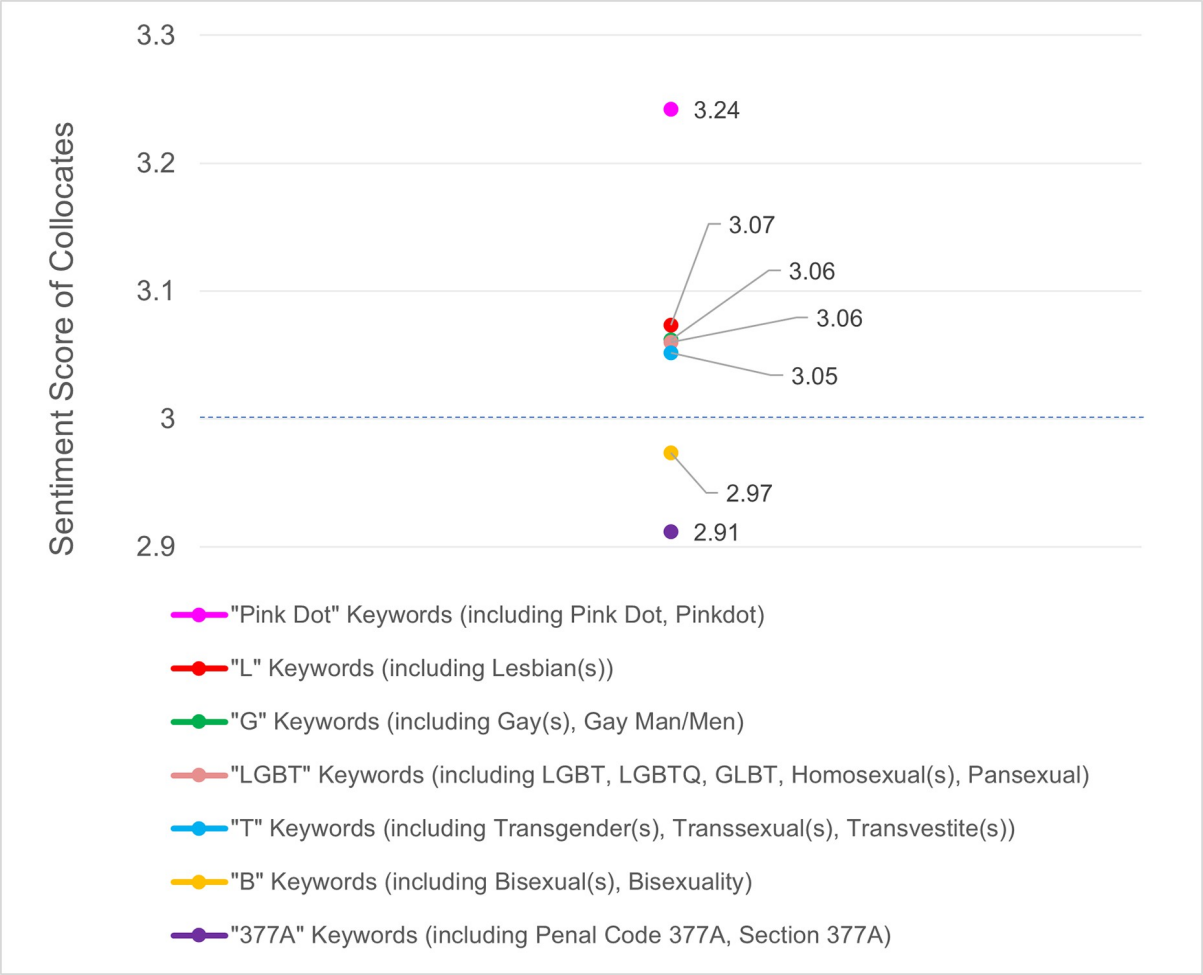
Sentiment score of LGBT categories in news media over a decade (2010–2020). Sentiment scores are assigned to collocates on a valence scale of 1–5, where 1 (very negative), 3 (neutral), 5 (very positive). Given that a neutral score is denoted by ‘3’, coverage about Pink Dot (the local pride event) was comparatively the most positive, followed by neutral scores for Lesbian, Gay, LGBT, Transgender. The only set of identity marker keywords that dipped slightly below the neutral threshold were Bisexual and 377A (the law against homosexual relations).

Overall, the sentiment score of LGBT collocates in mainstream media (Fig 3) hovered around the neutral score of ‘3’. Coverage about Pink Dot was comparatively the most positive (3.24), followed by neutral scores for Lesbian (3.07), Gay, LGBT (3.06), Transgender (3.05). The only set of keywords that dipped slightly below the neutral threshold were Bisexual (2.97) and 377A (2.91).

### Differing themes among LGBT and its component categories

We delved into the top collocates (descriptors) of the general keyword ‘LGBT’ in Singaporean mainstream media over the 2010–2020-decade, and discuss their thematic significance (Table 1). We further investigated the nuances of L, G, B, and T news narratives by means of their individual top collocates, providing evidence that the keywords *Lesbian*, *Gay*, *Bisexual*, and *Transgender* evinced different top collocations, suggesting divergence between mainstream depictions of these identities. These differing descriptors associated with each category—i.e., emergent unique standout themes specific to each identity marker—provide qualitative insights into how Singaporean news media construct and commonly portray L, G, B, and T stories.

**Table 1:** Key themes for LGBT and its component identities based on highest frequency descriptors over a decade (2010–2020).

Identity	Theme [unique standout themes highlighted (*)]	Sample Collocates (Descriptors)
**LGBT** Keywords: *LGBT*, *LGBTQ*, *GLBT*, *Homosexual(s)*, *Pansexual(s)*	**Legality**	*repeal*, *law*, *legal*, *penal*, *code*, *supreme*, *court*, *state*, *petition*, *legislation*, *constitutional*, *debate*, *bill*, *activist*, *advocate*, *marry*, *movement*, *protect*, *protection*, *civil*, *right*, *campaign*, *vote*
	**Objection**	*issue*, *objectionable*, *discrimination*, *ban*, *violence*, *assault*, *oppose*, *hateful*, *hate*, *refuse*, *discriminate*, *discretion*, *minority*
	**Support**	*allow*, *tolerate*, *accept*, *recognize*, *support*, *reasonable*, *openly*, *equality*, *pride*, *parade*, *equal*, *celebrate*, *march*, *promote*
	**Social Groups**	*conservative*, *Christian*, *church*, *catholic*, *religion*, *Muslim*, *school*, *military*, *cultural*, *culture*, *ethnicity*, *society*, *social*, *friend*, *parent*, *organization*, *young*, *student*
	**Identity**	*identify*, *expression*, *sexuality*, *status*, *love*, *sex*
**Lesbian** Keywords: *Lesbian(s)*	**Legality**	*law*, *legal*, *protection*, *activist*, *advocate*, *campaign*, *marry*, *allow*, *civil*, *wedding*, *sex*
	**Objection**	*discrimination*, *violence*, *ban*, *issue*, *minority*
	**Support**	*right*, *support*, *equality*, *openly*, *love*, *pride*, *parade*, *march*, *equal*, *celebrate*, *movement*, *raise*, *mardi*, *gras*
	**Social Groups**	*church*, *family*, *organization*, *association*, *school*, *youth*, *student*, *parent*, *network*, *national*, *coach*, *chairman*, *director*
	**Identity**	*identify*, *sexuality*, *human*, *female*, *mother*, *partner*, *daughter*, *feminist*, *young*, *transgendered*, *bi*
	Film, Sensuality, Violence (*)	*film*, *movie*, *character*, *show*, *scene*, *story*, *festival*, *feature*, *affair*, *kiss*, *rape*
**Gay** Keywords: *Gay(s)*, *Gay Man/Men*	**Legality**	*law*, *supreme*, *court*, *marry*, *activist*, *allow*, *civil*, *wedding*, *married*, *vote*, *legal*, *campaign*, *advocate*
	**Objection**	*discrimination*, *ban*, *oppose*, *refuse*, *homophobic*, *minority*, *issue*
	**Support**	*right*, *support*, *pride*, *parade*, *equality*, *love*, *movement*, *march*, *accept*, *celebrate*, *rainbow*, *equal*
	**Social Groups**	*catholic*, *Christian*, *church*, *religious*, *culture*, *Muslim*, *conservative*, *parent*, *society*, *friend*, *youth*, *student*
	**Identity**	*openly*, *identify*, *sexuality*, *sex*, *dating*, *partner*, *publicly*, *reveal*, *son*, *boy*, *male*, *young*
	Film (*)	*film*, *actor*, *character*, *scene*, *player*, *attitude*
	Violence, Death (*)	*hate*, *kill*, *attack*, *deny*, *death*, *shooting*, *nightclub*, *club*, *aids*
**Bisexual** Keywords: *Bisexual(s)*, *Bisexuality*	**Legality**	*law*, *activist*, *protection*, *campaign*, *advocate*, *organization*
	**Objection**	*discrimination*, *violence*, *minority*
	**Support**	*right*, *equality*, *love*, *promote*, *support*, *celebrate*, *protect*, *march*, *pride*, *parade*, *include*, *rainbow*, *accept*
	**Identity**	*questioning*, *question*, *identify*, *openly*, *sexuality*, *sex*, *straight*, *heterosexual*, *transgendered*, *asexual*, *student*, *young*, *youth*
	Population & Health Studies(*)	*diagnose*, *blood*, *research*, *risk*, *study*, *health*, *report*, *population*, *survey*, *reveal*, *percent*, *more*, *likely*, *experience*, *describe*, *compare*, *social*, *mental*
**Transgender** Keywords: *Transgender(s)*, *Transsexual(s)*, *Transvestite(s*	**Legality**	*law*, *court*, *legal*, *state*, *activist*, *advocate*, *access*, *bill*, *policy*, *recognize*, *education*, *administration*, *government*, *education*
	**Objection**	*discrimination*, *ban*, *violence*, *force*
	**Support**	*right*, *allow*, *support*, *protection*, *equality*, *protect*, *accept*, *pride*, *include*, *care*
	**Identity**	*identify*, *openly*, *announce*, *youth*, *young*, *school*, *child*
	Challenges(*)	*bathroom*, *restroom*, *require*, *military*, *serve*, *service*, *troop*, *prison*, *murder*, *kill*, *suicide*
	Transitioning(*)	*transition*, *treatment*, *surgery*, *change*, *birth*, *male*, *female*, *health-care*, *health*, *medical*, *experience*, *organization*, *center*, *social*, *worker*
	Film (*)	*actor*, *character*, *role*, *film*, *show*, *play*, *feature*, *athlete*, *model*

## Discussion

Findings suggested that over the 2010–2020-decade, local mainstream media coverage about the LGBT demographic steadily increased, which may be taken as a positive signal toward increasing visibility, representation, and discussion in local mainstream media. However, there was an evident sexuality bias, with the ‘G’ demographic dominating LGBT-related media coverage, leaving the ‘L’, ‘B’ and ‘T’ sub-groups comparatively under-mentioned. The fact that ‘G’ keywords were mentioned over 4 times more often than ‘L’ keywords may indicate a disparity in representation; a finding that is echoed in other LGBT media literature [3, 17].

We also found that sentiment scores about LGBT topics in Singapore’s mainstream news media over the 2010–2020 period remained relatively neutral on average. On a valence score of 1 to 5, with 1 being the most negative, 3 neutral, and 5 being the most positive, coverage about Pink Dot was comparatively the most positive (3.24), followed by neutral scores for Lesbian (3.07), Gay and LGBT (3.06), Transgender (3.05). The only set of keywords that dipped slightly below the neutral threshold were Bisexual (2.97) and 377A (2.91). The neutral scores were likely due to adhering to journalistic conventions of state media, delivering news as objectively and impartially as possible. Notably, however, *Pink Dot*, the local pride event, trended toward the most positivity, since collocates were often in relation to support and solidarity. A measure of caution may also be prudent given that of all LGBT identity markers, bisexuality skewed the most negative in comparison, suggesting that the portrayal of this demographic in mainstream media may benefit from careful messaging, to avoid skewing too negative. The topic of 377A trended toward the most negativity in terms of average collocate sentiment score, which was rather unsurprising given its incendiary nature: its news coverage often accompanied by conversations about *pushback*, *resistance*, and *rejection* from various sects.

### ‘LGBT’ themes

Thematic analyses suggested that overall, Singapore’s mainstream news media over the 2010–2020 decade overwhelmingly politicized the non-heterosexual identity: the lexical field of *legality* was frequently represented among the top collocations of ‘LGBT’ as a target keyword. The presence of words like *repeal*, *law*, *legislation*, *legal*, *penal code*, *supreme court*, *constitutional debate*, appearing in the context of whether 377A ought to be repealed, implies that LGBT issues have become intrinsically politicized and frequently collocated in contexts of legislation. Beyond that, words like *petition*, *activist*, *movement*, *campaign*, *civil*, *rights*, *protect*, *protection* suggest that LGBT narratives in mainstream media were also entwined with active campaigning on the grassroots level—reflective of a nation on the cusp of civil change [39]. In the context of mainstream media having the power to shape public attitudes and perceptions, it may be prudent to consider decoupling the connection between non-heterosexuality as a matter of legality; instead placing more focus on human interest features and away from overt politicization [20].

Top-frequency collocates also involved words in objection to, and in support of, LGBT causes. The former included pejoratives, running the gamut from the negatively-charged *objectionable*, *issue*, *oppose*, *refuse*, *discretion*, *discrimination*, *discriminate*, *minority*, to the outright vitriolic *violence*, *assault*, *hateful*, *hate*. The latter included positive words connoting to tolerance: *allow*, *accept*, *tolerate*, *recognize*, *reasonable;* and celebration of LGBT identities: *pride*, *parade*, *celebrate*, *equal*, *equality*, *support*, *openly*, *march*, *promote*. The theme of social sects, such as religion: *Christian*, *church*, *catholic*, *religion*, *Muslim*, and various social groups and relations, such as *conservative*, *school*, *military*, *organization*, *society*, *social*, *culture*, *cultural*, *ethnicity*, *friend*, *parent*; appeared in relation to numerous articles discussing support for, or opposition to, LGBT rights from various points of views. It was noteworthy that of top LGBT collocates, only a small handful were about self-expression and identity: *identify*, *expression*, *status*, *sexuality*, *sex*, *love*; suggesting that the state of LGBT representation and discussion in mainstream media was largely entrenched within the confines of legality compared to portrayals of self-expression—a trend representative of legal pragmatism within the confines of state-controlled media [40].

These findings provide food for thought about how LGBT issues have become synonymous with themes about polarized politicization. Given that media content, particularly those produced by state media and thus consumed by a wide audience, help sets the national agenda and contextual lens through which these groups are viewed, it may be prudent to assess whether such stories and themes should continue to percolate in the representation and viewing of this demographic.

### ‘Lesbian’ themes

Of the top-frequency collocates associated with the ‘Lesbian’ keyword, they shared thematic similarity with ‘LGBT’ collocates in the semantic fields of legality, objection and support, echoing similar polarizing themes. Beyond that, a new set of collocates emerged, particularly about the theme of identity expression, with the presence of more feminine relational terms like *female*, *mother*, *daughter*, *feminist*. Notably, the emergence of a new theme- largely about film- *movie*, *character*, *show*, *scene*, *story*, *festival* (i.e., *film festival*) and *feature* (i.e., *feature film*) suggests increasing representation of lesbian content in films and television, with these features discussed in mainstream news media contexts. The presence of words denoting sensuality and sexual violence: *affair*, *kiss*, *rape* suggest that the contexts of such stories may have had an added element of sensationalism.

### ‘Gay’ themes

In a similar vein, top-frequency collocates associated with ‘Gay’ as a target keyword contained common themes of legality issues and equal parts objection (*homophobic*, *discrimination*, *oppose*) and support (*celebrate*, *equality*, *love*, *movement*) and commonly occur in relation to social—often either *religious* or *conservative*—sects. Collocates about film were present (*character*, *actor*, *scene*, *film*); from movie coverage about the demographic, or news about certain celebrities who have publicly come out as gay. Unique themes that emerged included the presence of violence: *hate*, *kill*, *attack*, *death*, *shooting*; in relation to articles published about hate crimes enacted upon gay men (for instance, *nightclub* and *club* in relation to a terrorist attack on a gay nightclub in Florida, 2016 [41]. Notably, *aids* was a top collocate for this group; owing to the upsetting cultural association linking this identity marker with AIDS, in spite of the possibility of heterosexual transmission [42]. Overall, these negative words have become collocated with this subgroup, suggesting that more may be done to decouple negativity from their stories—or to include more uplifting news into the mix.

### ‘Bisexual’ themes

Of the top-frequency collocates associated with the ‘bisexual’ identity marker, the themes of legality, objection and support were similarly present. Uniquely, collocates associated with identity were less affirming compared to the latter categories—containing words relating to uncertainty (*questioning*, *question*) and including terms from opposite ends of the dichotomized spectrum of sexuality (*straight*, *heterosexual*; *asexual*). The presence of these collocates is emblematic of this sexuality often being comparatively overlooked in a mediatized version of bisexual erasure [43]. Interestingly, collocates about population studies were common for this group (*research*, *study*, *report*, *population*, *survey*, *percent*), and relating to the medical field (*diagnose*, *blood*, *mental*, *health*)—from articles and reports that include this target group in nation-wide survey results.

### ‘Transgender’ themes

Of the top-frequency collocates associated with the ‘transgender’ demographic, common themes of legality, objection and support, and identity were present. Unique collocational themes included myriad challenges faced by this community: for instance, collocates *bathroom* and *restroom* are indicative of the struggle many transgender individuals face in using public bathrooms aligned to their gender identity, and pushback from detractors who cite instances of misuse [44].

Other collocates like *military service* and *troop* appeared in relation to a published story from 2016 [45]: a Singaporean transgender woman sought asylum in the UK to avoid serving reservist time in the military, as she was by then living as a woman and uncomfortable with being in the same bunk as multiple other men. Such news stories gained local traction due to the presence of unique circumstances: transgender women are exempt from performing national service in Singapore only if they have undergone gender-affirming surgery. Similarly, the collocate *prison* appeared, in relation to the circulation of several stories related to assault of a partner; and a story about a transgender inmate—who had an identification card aligned with his gender identity and had not undergone gender-affirming surgery—sentenced to a male prison; sparking discussion on whether he would potentially be harassed in prison [46].

Other collocates like *murder* and *suicide* made the list due to the increased likelihood of transgender youths and adults being prone to suicide ideation and attempts compared to other demographics [47]. Collocates about medically transitioning (*treatment*, *surgery*, *medical*, *experience*, *change*) speak to the experience of pursuing and undergoing sex reassignment surgery. Collocates like *social*, *worker*, *center* were also collocated with this group, against the backdrop of strong social services like the T project, Singapore’s first and only shelter for homeless transgender women [48].

Lastly, film-related collocates (*character*, *role*, *film*, *show*, *play*, *feature*) and professions (*actor*, *athlete*, *model*) suggested greater representation for this demographic; though the presence of collocates like *athlete* due to debates surrounding transgender athletes, with questions on whether elevated testosterone or average strength may give athletes unfair advantages in competitive sporting events [49].

These findings, taken together, suggest that even among each sub-group (L, G, B, T), certain groups occupy the discursive margins of this already marginalized demographic. Each sub-group contained different issues that may have become salient as top collocates through repetition via media association and frequent reporting—certain themes may have unwittingly been framed as strongly associated with specific groups. These findings suggest that more nuance and a greater diversity of stories may revitalize conversations surrounding these sub-demographics, rather than monolithically and one-dimensionally representing such identities.

This study acknowledges several limitations. First, our corpus is limited to news articles written in English. Further study may be required to elucidate differences in representation, particularly across media written in different languages. Second, our use of big data is a double-edged sword: while we have provided societal-level insights, such socially divisive issues also require nuanced interpretations afforded by close reading (e.g., discourse studies that monitor subtle shifts in tone across individual articles written across a decade). Hence, this study’s conclusions are meant to be interpreted as a large-scale overview of prominent trends in media representation that supplement nuanced analysis of the LGBT discoursal landscape; finer-grained analysis across smaller time-scales may be conducted in follow-up studies. For instance, our collocational methodology may be replicated on a year-by-year level to identify granular stories or themes that were most dominant within that year, for each identity category.

## Conclusions

This exploratory study found three main insights about LGBT representation in Singapore mainstream media from 2010 to 2020. First, overall coverage size was significantly following a positive linear increase. However, Gay identity keywords appeared in the corpus more frequently than Lesbian, Bisexual, and Transgender keywords. Second, collocate sentiment scores for *Pink Dot* were most positive; *Lesbian*, *Gay*, *LGBT*, *Transgender* were neutral; *Bisexual* and *377A* dipped slightly negative. Third, top LGBT collocates occupied the lexical field of legality; uniquely, ‘Lesbian’ collocates related to sensationalized cinema; ‘Gay’ about hate crimes; ‘Bisexual’ about population surveys; ‘Transgender’ about challenges like transitioning, alienation, and suicide.

Practically, this study presents a replicable framework and lens through which the methodology may be repeated across other Southeast Asian nations that have inherited similar anti-homosexuality laws from old colonial rule. Significantly, these results present a large-scale overview of state media from a large swathe of news articles spanning a decade and provide key insights into how LGBT identities and issues were saliently portrayed in mainstream media.
